# Effects of Rhizosphere Bacteria on Strawberry Plants (*Fragaria × ananassa* Duch.) under Water Deficit

**DOI:** 10.3390/ijms231810449

**Published:** 2022-09-09

**Authors:** Dominika Paliwoda, Grzegorz Mikiciuk, Małgorzata Mikiciuk, Anna Kisiel, Lidia Sas-Paszt, Tymoteusz Miller

**Affiliations:** 1 Department of Horticulture, Faculty of Environmental Management and Agriculture, West Pomeranian University of Technology in Szczecin, Słowackiego 17, 71-434 Szczecin, Poland; 2 Department of Bioengineering, Faculty of Environmental Management and Agriculture, West Pomeranian University of Technology in Szczecin, Słowackiego 17, 71-434 Szczecin, Poland; 3 Institute of Marine and Environmental Sciences, University of Szczecin, Wąska 13, 71-415 Szczecin, Poland; 4 Polish Society of Bioinformatics and Data Science BIODATA, Popiełuszki 4c, 71-214 Szczecin, Poland; 5 Department of Microbiology and Ryzosphere, The National Institute of Horticultural Research, Pomologiczna 18, 96-100 Skierniewice, Poland

**Keywords:** fluorescence of chlorophyll “a”, bacterial counts, drought stress, PGPR, strawberry

## Abstract

Due to the observed climate warming, water deficiency in soil is currently one of the most important stressors limiting the size and quality of plant crops. Drought stress causes a number of morphological, physiological, and biochemical changes in plants, limiting their growth, development, and yield. Innovative methods of inducing resistance and protecting plants against stressors include the inoculation of crops with beneficial microorganisms isolated from the rhizosphere of the plant species to which they are to be applied. The aim of the present study was to evaluate 12 different strains of rhizosphere bacteria of the genera *Pantoea*, *Bacillus*, *Azotobacter*, and *Pseudomonas* by using them to inoculate strawberry plants and assessing their impact on mitigating the negative effects of drought stress. Bacterial populations were assessed by estimates of their size based on bacterial counts in the growth substrate and with bioassays for plant growth-promoting traits. The physiological condition of strawberry plants was determined based on the parameters of chlorophyll fluorescence. The usefulness of the test methods used to assess the influence of plant inoculation with rhizosphere bacteria on the response of plants growing under water deficit was also evaluated. A two-factor experiment was performed in a complete randomization design. The first experimental factor was the inoculation of plant roots with rhizosphere bacteria. The second experimental factor was the different moisture content of the growth substrate. The water potential was maintained at −10 to −15 kPa under control conditions, and at −40 to −45 kPa under the conditions of water deficit in the substrate. The tests on strawberry plants showed that the highest sensitivity to water deficiency, and thus the greatest usefulness for characterizing water stress, was demonstrated by the following indices of chlorophyll “a” fluorescence: F_M_, F_V_, F_V_/F_M_, PI, and Area. Based on the assessment of the condition of the photosynthetic apparatus and the analysis of chlorophyll “a” fluorescence indices, including hierarchical cluster analysis, the following strains of rhizosphere bacteria were found to have favorable effects on strawberry plants under water deficit: the *Bacillus* sp. strains DLGB2 and DKB26 and the *Pantoea* sp. strains DKB63, DKB70, DKB68, DKB64, and DKB65. In the tests, these strains of *Bacillus* sp. exhibited a common trait—the ability to produce siderophores, while those of *Pantoea* sp. were notable for phosphate mobilization and ACCD activity.

## 1. Introduction

Due to the observed climate warming, water deficiency in soil is currently one of the most important stressors limiting the size and quality of plant crops. On a global scale, drought affects one-third of soils [1]. In modern agriculture, the unfavorable impact of commonly used pesticides and chemical fertilizers on limiting the microbiological diversity of soils also constitutes a major problem [2].

Soil water deficit may lead to, inter alia, a reduction in the efficiency of photosynthesis through its influence on the functioning of the stomata and on the process of accumulation and transport of assimilates [3,4]. Stress initiates the operation of mechanisms of scattering the excess absorbed light energy, one of them being the fluorescence of chlorophyll “a”. Hence, research methods and techniques based on this phenomenon are among the most sensitive of those used to identify stress states in plants. They are also non-invasive and rapid. They are considered to be useful methods for the assessment of plant tolerance to environmental stressors [5].

In order to manage water most rationally, both for economic and ecological reasons, new solutions are sought to reduce the impact of water deficit on crops. Innovative methods of inducing resistance and protecting plants against stressors include the inoculation of crops with beneficial microorganisms isolated from the rhizosphere of the plant species to which they are to be applied. Groups of rhizosphere microorganisms and their interactions are the subject of research aimed at determining their influence on the growth, yield, and protection of plants [6,7,8]. These organisms colonize the roots of plants and induce growth and immune processes in them, which can contribute to the neutralization or minimization of the impact of stresses caused by, for example, water deficiency [9,10,11,12], high temperature [13,14], low temperature [15], salinity [16,17], soil contamination with heavy metals [18,19], or biotic factors (pathogens) [20,21]. Among free-living bacteria are Plant Growth Promoting Rhizobacteria (PGPR), which have been utilized for improving water and nutrient uptake and abiotic and biotic stress tolerance [22,23]. Among the mechanisms involved, there are: phosphate mobilization, ammonia production, organic acids production, nitrogenase activity, biofilm formation, the production of siderophores, exopolysaccharides, phytohormones (including indole-3-acetic acid—IAA), and 1-aminocyclopropane-1-carboxylate (ACC) deaminase activity [24,25,26,27]. Many studies have shown enhanced stress tolerance in plants through inoculation with PGPR. PGPRs secrete ACC deaminase, which destroys an ethylene precursor ACC, protecting plants against drought stress [28,29,30,31]. It has been reported that auxin-producing bacteria also protect plants from stress by increasing root length, the number of root tips, root surface area, and total plant biomass. As a result, they increase the uptake of water and nutrients and stimulate the synthesis of ACC deaminase [32,33]. Bacteria promoting plant growth and yielding under both normal and stressful conditions include, among others: *Azospirillum*, *Arthrobacter*, *Bacillus*, *Enterobacter*, *Pseudomonas*, *Rhizobium*, *Pantoea*, and *Azoarcus* [2,34,35,36].

The strawberry is an economically important berry species [37] that is characterized by high sensitivity to water deficiency [38,39,40]. This species is therefore a very good object of research on the effects of factors that can potentially relieve drought stress, including the effectiveness of inoculating plants with plant growth-promoting bacteria.

The aim of the present study was to evaluate 12 strains of rhizosphere bacteria of the genera *Pantoea*, *Bacillus*, *Azotobacter*, and *Pseudomonas* by using them to inoculate strawberry plants and assessing their impact on mitigating the negative effects of drought stress. Bacterial populations were assessed by estimating their size based on bacterial counts in the growth substrate and with bioassays for plant growth-promoting traits. The physiological condition of strawberry plants was determined based on the parameters of chlorophyll fluorescence. The research results may be used for selecting PGPR strains with the highest anti-stress potential to reduce the potential adverse effects of water deficit on the growth and yielding of plants.

The usefulness of the test methods used to assess the influence of plant inoculation with rhizospheric bacteria on the response of plants growing under water deficit was also evaluated.

## 2. Results

### 2.1. Bioassays for Plant Growth Promoting Traits

All of the strains reported on in the present study exhibited PGP traits in the biochemical assays. Among the tested strains, only two (*Azotobacter* sp. AJ 1.1 and *Pseudomonas* sp. PJ 1.2) manifested all of the key traits of PGPR. Some of these strains were capable of biosynthesizing indolic compounds in the presence of L-tryptophan. Indole 3-acetic acid was produced by *Azotobacter* sp. AJ 1.1, *Pseudomonas* sp. PJ 1.2, and tree strains belonging to the *Pantoea* species: DKB 64, DKB 65, and DKB 70. Seven strains tested are very promising for the production of siderophores, iron chelating compounds. These included the strains of *Azotobacter* sp. AJ 1.1, *Pseudomonas* sp. PJ 1.2, and all those belonging to *Bacillus* sp. Among the tested strains, seven showed the ability to convert insoluble inorganic phosphorus (P) compounds, such as tricalcium phosphate, to bioavailable forms (*Azotobacter* sp. AJ 1.1, *Pseudomonas* sp. PJ 1.2, and all those belonging to *Pantoea* sp.). Ten strains expressed the ACC deaminase (ACCD) activity at levels ranging from 62.5 to 8306 nmol α-KB·mg protein^−1^·h^−1^. The highest ACCD activity (8306.25 nmol α-KB·mg protein^−1^·h^−1^) was exhibited by *Azotobacter* sp. AJ 1.1, followed by *Pseudomonas* sp. PJ 1.2 (6287.5 nmol α-KB·mg protein^−1^·h^−1^), two strains of *Pantoea* sp.: DKB 65 (4757 nmol α-KB·mg protein^−1^·h^−1^) and DKB 68 (3862.25 nmol α-KB·mg protein^−1^·h^−1^). The ACCD activity of the *Bacillus* sp. strains was expressed at levels ranging from 2946.75 (DKB 58) to 853 (DKB 26) nmol α-KB·mg protein^−1^·h^−1^. Two strains belonging to *Bacillus* sp. (DLGB 2 and DLGB 3) showed no ACCD activity (Table 1).

### 2.2. Bacterial Counts in Substrate

**Figure 1:**
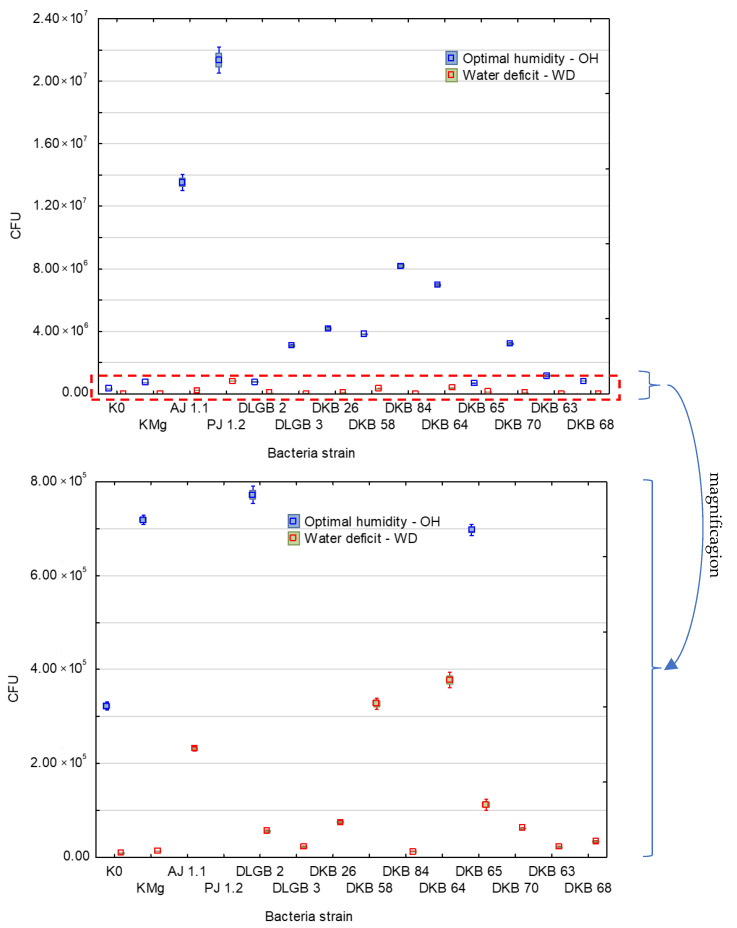
Bacterial counts in growth substrate after inoculation with rhizosphere bacteria under different levels of substrate moisture.

In the present study, in the case of optimum moisture content, the lowest numbers of bacteria were observed in the control variants, i.e., K0 (3.2 × 10^5^ CFU/g of substrate) and KMg with magnesium sulfate (7.2 × 10^5^ CFU/g of substrate). Only one strain, *Pantoea* sp. DKB 63, caused a reduction in the number of bacteria in relation to KMg (to the level of 1.2 × 10^5^ CFU/g of substrate), while the remaining strains caused an increase in the number of bacteria in the soil. The highest numbers of bacteria were observed after inoculation with *Pseudomonas* sp. PJ 1.2 (2.1 × 10^7^ CFU/g of substrate) and *Azotobacter* AJ 1.1 (1.3 × 10^7^ CFU/g of substrate). In the case of moisture deficit in the substrate, a negative correlation was observed for all the variants at the level of −0.46, which was manifested in a considerable reduction in the total number of bacteria in the soil. The lowest values of CFU per g of substrate were recorded for the controls K0 (8.5 × 10^3^) and KMg (1.3 × 10^4^). After bacterial inoculation, the highest numbers of bacteria were recorded for the strains of *Pseudomonas* sp. PJ 1.2 (8.2 × 10^5^ CFU/g of substrate), *Pantoea* sp. DKB 64 (3.8 × 10^5^ CFU/g of substrate), and *Bacillus* sp. DKB 58 (3.3 × 10^5^ CFU/g of substrate) (Figure 1).

### 2.3. Chlorophyll Fluorescence

**Figure 2:**
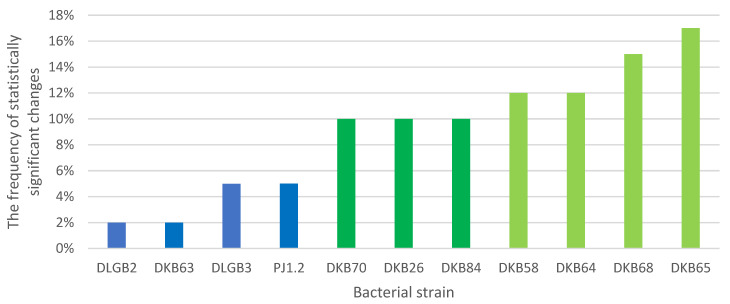
Frequency of statistically significant changes in the indices of chlorophyll “a” fluorescence in strawberry as a result of inoculation with rhizosphere bacteria, between optimal soil moisture and water deficit conditions.

The study analyzed the frequency of statistically significant differences in the values of the determined parameters of chlorophyll “a” fluorescence in strawberry, between the control variants (K0 and KMg) and the variants differing in the bacterial strains used for inoculation, regardless of the substrate moisture level. The analysis revealed four inoculation strains that accounted for 56% of the variability of chlorophyll “a” fluorescence indices; they were: *Bacillus* sp. DKB 58, *Pantoea* sp. DKB 64, *Pantoea* sp. DKB 65, and *Pantoea* sp. DKB 68. It should be noted that after including the next three strains—i.e., *Bacillus* sp. DKB 84, *Pantoea* sp. DKB 70, and *Bacillus* sp. DKB 26—the seven strains together accounted for 85% of the variability of the determined indices of chlorophyll “a” fluorescence, regardless of the substrate moisture level (Figure 2).

**Figure 3:**
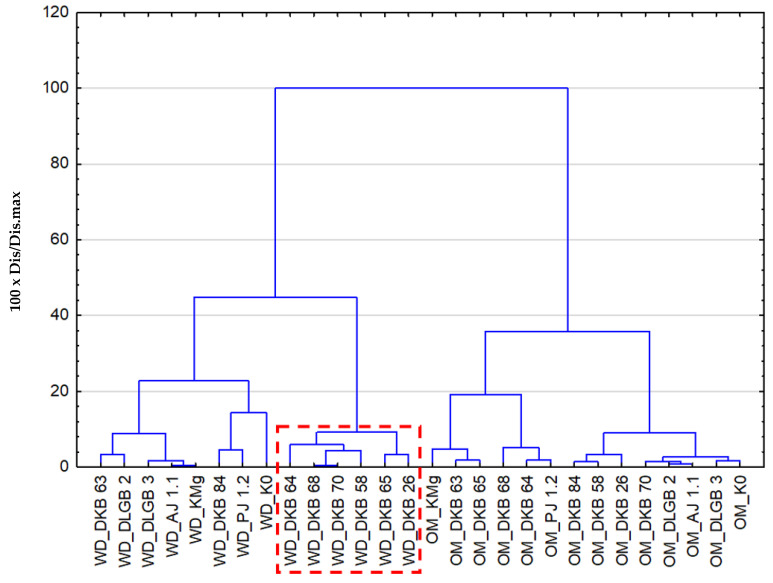
Cluster analysis showing the similarities of the variability of chlorophyll “a” fluorescence indices, depending on the different substrate moisture levels and inoculation with rhizosphere bacteria, where: OM—optimal moisture, WD—water deficit.

The hierarchical cluster analysis revealed three important clusters: cluster I and cluster II represented the similarity of the variability of chlorophyll “a” fluorescence indices under the conditions of water deficit in the substrate, and cluster III was a complex cluster encompassing the variability under optimal moisture levels. Cluster I included strains whose variability coincided with the changes in the control samples K0 and KMg. Cluster II, which was mainly responsible for the variability of these traits caused by different moisture levels, included the strains: *Pantoea* sp. DKB 64, *Pantoea* sp. DKB 68, *Pantoea* sp. DKB 70, *Bacillus* sp. DKB 58, *Pantoea* sp. DKB 65, and *Bacillus* sp. DKB 26. Cluster III showed the dependence of the variability of the effect of the inoculation strains on the magnitude of the determined indices of chlorophyll “a” fluorescence in a manner dependent on K0 and KMg. The most important feature observed in the experiment is the total distinctiveness in terms of the tested fluorescence indices between the optimal soil moisture and water deficit (Figure 3).

**Figure 4:**
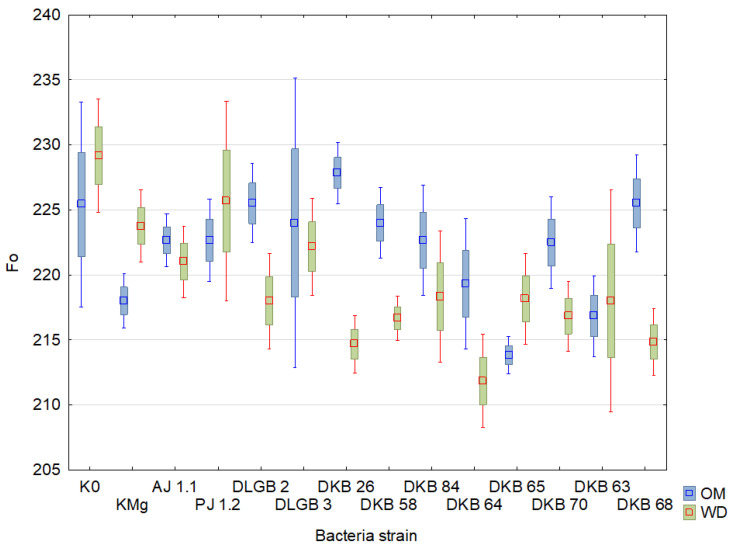
F_0_ in the leaves of strawberry plants inoculated with rhizosphere bacteria growing under different substrate moisture levels, where: OM—optimal moisture, WD—water deficit.

**Figure 5:**
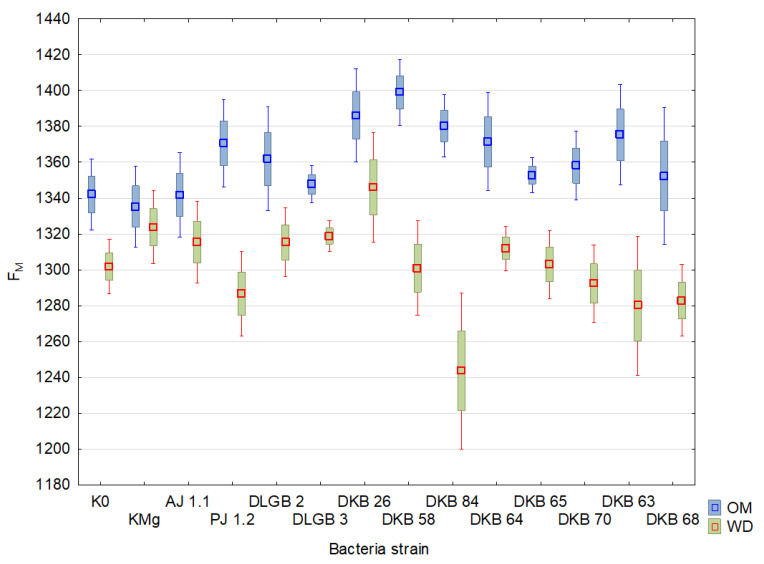
F_M_ in the leaves of strawberry plants inoculated with rhizosphere bacteria growing under different substrate moisture levels, where: OM—optimal moisture, WD—water deficit.

**Figure 6:**
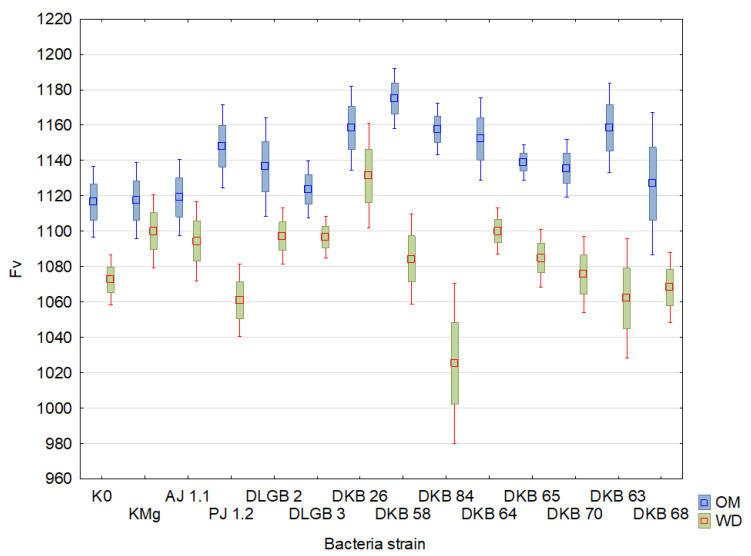
F_V_ in the leaves of strawberry plants inoculated with rhizosphere bacteria growing under different substrate moisture levels, where: OM—optimal moisture, WD—water deficit.

**Figure 7:**
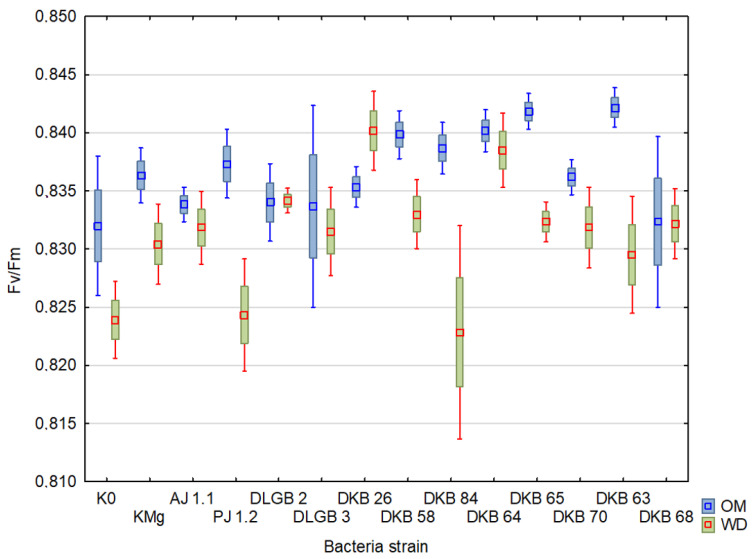
F_V_/F_M_ in the leaves of strawberry plants inoculated with rhizosphere bacteria growing under different substrate moisture levels, where: OM—optimal moisture, WD—water deficit.

**Figure 8:**
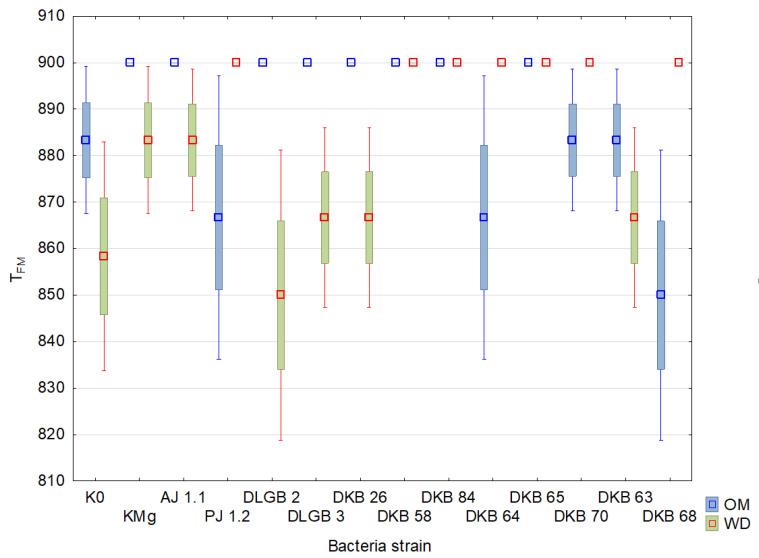
T_FM_ in the leaves of strawberry plants inoculated with rhizosphere bacteria growing under different substrate moisture levels, where: where: OM—optimal moisture, WD—water deficit.

**Figure 9:**
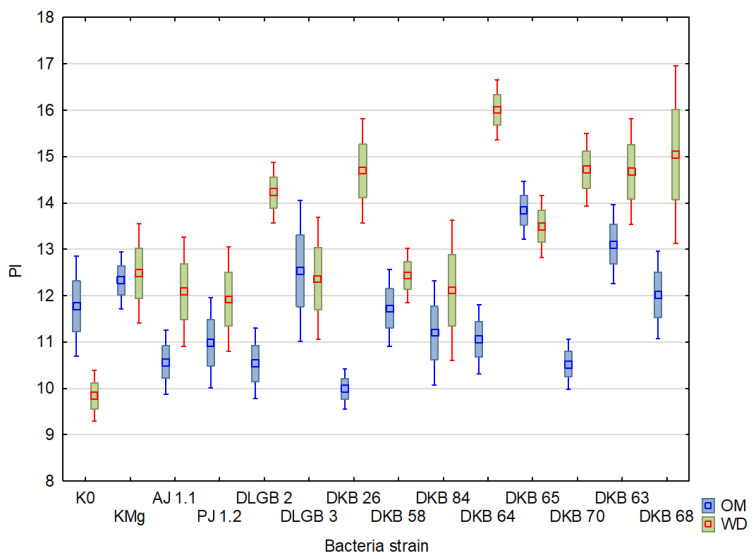
PI in the leaves of strawberry plants inoculated with rhizosphere bacteria growing under different substrate moisture levels, where: OM—optimal moisture, WD—water deficit.

**Figure 10:**
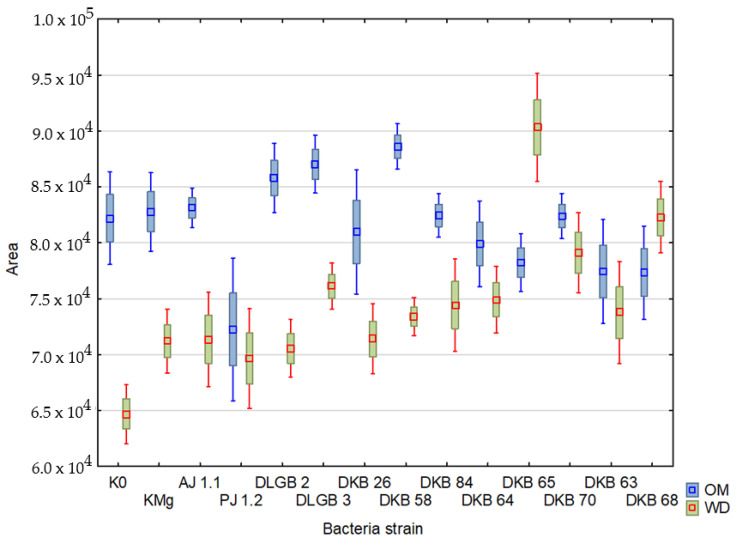
‘Area’ parameter in the leaves of strawberry plants inoculated with rhizosphere bacteria growing under different substrate moisture levels, where: OM—optimal moisture, WD—water deficit.

The results of the descriptive statistics of the conducted experiment are presented in Table 2 and Table 3 and Figure 4, Figure 5, Figure 6, Figure 7, Figure 8, Figure 9 and Figure 10. The results of the analysis of variance are shown in Table 4. The F_0_ coefficient varied in the range from 193 to 376 in the conditions of optimal moisture, reaching a CV of 9.5%, while in the conditions of water deficit, the coefficient was between 159 and 373 and CV = 11%. The lowest values were recorded when inoculated with the *Bacillus* sp. DLGB 2 (OM) and *Pantoea* sp. DKB 63 (WD) strains, and the highest when inoculated with the *Bacillus* sp. DLGB 2 (OM) and *Pantoea* sp. DKB 63 (WD) strains (Table 2, Figure 4).

The F_M_ coefficient averaged 1258 and varied in the range from 984 to 1487 under optimal moisture conditions, reaching a CV of 6.5%, while under water deficiency conditions, the coefficient was between 779 and 1464 and CV = 7%. The lowest values were recorded in the control K0 (OM) and when inoculated with the *Pantoea* sp. DKB 63 (WD) strain, and the highest when inoculated with the *Pseudomonas* sp. PJ 1.2 (OM) strain and KMg (WD) (Table 4, Figure 5).

The F_V_ coefficient averaged 1032 and varied from 736 to 1259 under optimal moisture conditions, reaching a CV of 7.5%, while under water deficit, the coefficient was between 554 and 1237 and CV = 9.8%. The lowest values were recorded in the control K0 (OM) and when inoculated with the *Pantoea* sp. DKB 63 (WD) strain, and the highest when inoculated with the *Pseudomonas* sp. PJ 1.2 (OM) strain and KMg (WD) (Table 4, Figure 6).

The F_V_/F_M_ ratio averaged 0.82 and fluctuated in the range from 0.64 to 0.85 under optimal moisture conditions, reaching a CV of 4.1%, while under water deficit, the ratio was between 0.60 and 0.85 with CV = 3.6%. The lowest values were recorded at inoculation with the *Bacillus* sp. DLGB 2 (OM) and *Pantoea* sp. DKB 63 (WD) strains, and the highest in inoculation with the *Bacillus* sp. DLGB 3 (OM) and *Pantoea* sp. DKB 70 (WD) strains (Table 4, Figure 7).

The PI coefficient was on average 10.77 and varied in the range from 0.51 to 17.72 under optimal moisture conditions, reaching a CV of 34%, while under water deficit, the coefficient was between 0.27 and 23.97 with CV = 29.8%. The lowest values were recorded during inoculation with the strains *Bacillus* sp. DLGB 2 (OM) and *Pantoea* sp. DKB 63 (WD), and the highest when inoculated with *Bacillus* sp. DLGB 3 (OM) and *Pantoea* sp. DKB 68 (WD) (Table 2, Figure 9).

The Area coefficient averaged 69.568 and varied in the range from 27,371 to 102,457 under optimal moisture conditions, reaching a CV of 17%, while under water deficit, the coefficient was between 17,269 and 107.411, CV = 19% The lowest values were recorded when inoculating with the *Bacillus* sp. DLGB 3 strains (OM) and *Pantoea* sp. DKB 63 (WD), and the highest at KMg (OM) and *Pantoea* sp. DKB 70 (WD) (Table 2, Figure 10).

## 3. Discussion

Members of the *Bacillus*, *Pseudomonas*, and *Azotobacter* genera of bacteria have been extensively reported as plant growth enhancers [41,42,43,44,45]. Those of the genus *Pantoea*, although usually known as plant pathogens, have been reported in some studies to include strains with plant growth-promoting capabilities [32,46,47]. Indole-3-acetic acid (IAA), a plant hormone, is a natural auxin produced by rhizobacteria; as one of the phytohormones, IAA can act as a reciprocal signaling molecule in microbe-plant interactions [48]. Siderophore-producing PGPRs increased the Fe(III) ion supply to plants in the rhizosphere and are, therefore, known to enhance plant growth and crop productivity [49]. The ability to produce a clear zone around the bacterial colony implies that the bacteria can solubilize mineral phosphorus in the rhizosphere [50]. Plant growth-promoting rhizobacteria which possess the enzyme 1-aminocyclopropane-1-carboxylate (ACC) deaminase facilitate plant growth and development by decreasing ethylene levels, inducing a reduction in drought stress in plants [51]. The production of key traits for PGPR strains was found among many representatives of the bacteria used in this work and they were also tested in the context of drought [52,53,54,55].

There are a number of reports on the mitigation of adverse effects of drought by the addition of PGP microbes [56,57]. The most often used for this purpose are strains of the genus *Bacillus* [27,58,59], with those of *Pantoea* [60] and *Azotobacter* [61] used to a lesser extent.

There are not many reports on the changes in the numbers of microorganisms under drought conditions. For example, culture-based methods (intact grassland monoliths from natural habitats) have indicated that microbial physiological response was modulated by moisture content. The highest numbers of bacteria were observed with wetted treatments consistently being over 10^8^ CFU/g of substrate, and there was up to a 40-fold reduction in CFU in dried treatments, compared with the continually wetted treatments [62]. Similar observations were noted by Omar et al. [63], where the decrease in the numbers of bacteria, after they had been subjected to drought stress, varied according to the rice genotype from 9.3% to 20%. When comparing the survival of *Azospirillum* strains under stressful conditions in maize cultivation, Ilyas et al. [64] observed a decrease in the number of these bacteria by 40%.

In this study, the smallest decrease in the number of bacteria in the case of moisture deficit, compared with optimum moisture content, was observed following inoculations with the strains *Pantoea* sp. DKB 65 (approx. 5 times), *Bacillus* sp. DKB 58 (approx. 11 times) and *Bacillus* sp. DLGB 2 (approx. 13 times).

Chlorophyll fluorescence can be considered the basic indicator for the analysis of the relationship between photosynthesis and plant growth environment. It is used, inter alia, in studies on the response of various plant species to stressors [65,66,67]. Under the influence of water deficiency, the probability of PSII damage increases, manifested by a reduced photosynthetic efficiency and an increase in the dissipation of absorbed energy in the form of non-photochemical quenching [68].

The study did not show any influence of the experimental factors or their interactions on the values of the parameters F_0_ and T_FM_. The F_0_ index indicates the amount of loss in the excitation energy during its transmission from the antennas to the PSII active center. T_FM_ is the time to peak fluorescence. In plants growing under the conditions of water deficiency, the study found a decrease in the parameters F_M_, F_V_, F_V_/F_M_, and also PI and Area, which confirmed the presence of the state of stress. This is indicative of a significant impact of drought on the state of the photosynthetic apparatus of the tested plant species, a lower quantum yield of PSII, the inability to reduce all electron acceptors, and the occurrence of energy losses in the form of heat. According to Xu [69] and Angelini et al. [70], the ratio F_V_/F_M_ is considered a reliable indicator of the photochemical activity of the photosynthetic apparatus, which determines the potential efficiency of PSII. When this ratio becomes lower, it proves that the plant has been exposed to a stressor. A reduction in the F_V_/F_M_ value due to drought, which was demonstrated in our study, has also been observed in other species such as: *Lycopersicon esculentum* Mill. [71], *Viburnum tinus* L. [72], and *Vigna inguiculata* L. Walp. [73]; in the case of the latter—as a result of prolonged water deficiency stress. There are also reports of the relatively low sensitivity of the F_V_/F_M_ ratio to water deficiency, which has been found in various species: *Phaseolus vulgaris* L. [74], *Glycine max.* (L.) Merr [75], *Secale cereale* L. [76], and also *Fragaria × ananassa* Duch. [77]. The PI index is considered a reliable parameter for assessing the tolerance of plants to abiotic stresses and is an indicator of the efficiency of the PSII system [78]. Our study showed a significant decrease in the value of this index by 10.6% due to water deficiency. Similar results of research on the impact of drought stress on the PI parameter in rye had been reported by Czyczyło-Mysza and Myśków [76].

There is evidence that by inducing various mechanisms, such as the production of phytohormones (IAA, cytokinins, ABA), the production of bacterial exopolysaccharides (EPS), and the synthesis of the ACC deaminase enzyme, rhizospheric microorganisms can promote plant growth under drought stress [79,80]. In the present study, the inoculation with the tested strains of rhizospheric bacteria did not affect the value of the parameters F_M_, F_V_, and F_V_/F_M_, neither under the conditions of drought nor under optimal substrate moisture (Figure 5, Figure 6 and Figure 7). Barnawal et al. [81], however, had shown, under water deficit, a beneficial effect of the inoculation of wheat with the *Bacillus subtilis* LDR2 strain on the efficiency of the PSII system, which was manifested by an increase in the F_V_/F_M_ ratio. Similarly, Khan et al. [82] had shown an increase in F_V_/F_M,_ as a result of using a consortium of the bacteria *Bacillus subtilis*, *Bacillus thuringiensis*, and *Bacillus megaterium* in two varieties of *Cicer arietinum* L. contrasting for drought tolerance, growing under water deficit. This increase was particularly evident in the drought-sensitive variety.

In the case of the plants growing under water deficit, the present study showed an increase, in comparison with the control, in the PSII vitality index due to inoculation with the *Bacillus* sp. strains, DLGB2 and DKB26, as well as the *Pantoea* sp. strains, DKB63, DKB70, DKB68, and DKB64. There was also evidence of a beneficial effect of inoculation with the *Pantoea* sp. strains, DKB70, DKB68, and DKB65 on the increase, relative to the control, in the value of the Area parameter in the strawberry plants growing under the conditions of water deficit in the substrate. This may indicate an increase in the efficiency of electron transport from the reaction centers to the plastoquinones. A synergistic effect of the *Pseudomonas* strains in the production of ACC deaminase, auxin synthesis, the ability of mineral phosphate solubilizing, and the production of siderophores, has been found to significantly improve the yield-related traits of sweetcorn under the limited availability of irrigation water. Moreover, there was an increase in the F_V_/F_M_ index and a decrease in the F_0_ index after inoculation with bacteria [83].

## 4. Materials and Methods

### 4.1. Location of the Experiment, Plant Material, and Growth Conditions

**Figure 11:**
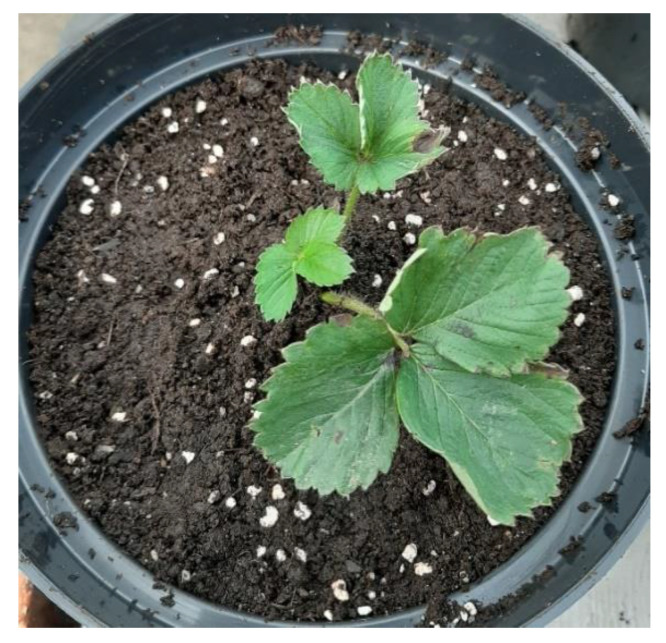
The plantlet of the strawberry cv. Polka.

The experiment was conducted in a greenhouse, located at the West Pomeranian University of Technology in Szczecin (53°25′ N, 14°32′ E, 25 m a.s.l., sub-zone 7a USDA). On 5 October 2020, plantlets of the strawberry cultivar Polka (Strawberry plant nursery J.G. Mendyk, Koronowo, Poland) were planted. Strawberry cv. Polka is one of the tastiest medium-late varieties of strawberries. The fruits are medium size, spherical, heart-shaped, or broad-conical-shaped. They have uniformly red to dark red skin with a slight gloss. The plants are moderately strongly growing. ‘Polka’ is resistant to frost, leaf scorch disease, as well as powdery mildew, and are susceptible to common gray mold. Plantlets (BBCH13) were planted individually into black round PVC pots with a diameter of 19 cm, and a capacity of 3.0 dm^3^, filled with peat substrate (Substral Osmocote, Evergreen Garden Care Poland Sp. Z o. o.), and mixed with perlite (at 15:1)—Figure 11. The substrate (pH 6.2) was enriched with a 2 g·dm^−3^ mixture of Osmocote NPK 15-09-09 and Plant Starter NPK 10-52-10. No additional fertilization was applied during plant vegetative growth. The plants were cultivated under natural day/night conditions, 8h:16h day: night, without artificial lighting, at 17–20 °C.

### 4.2. Experimental Factors

**Figure 12:**
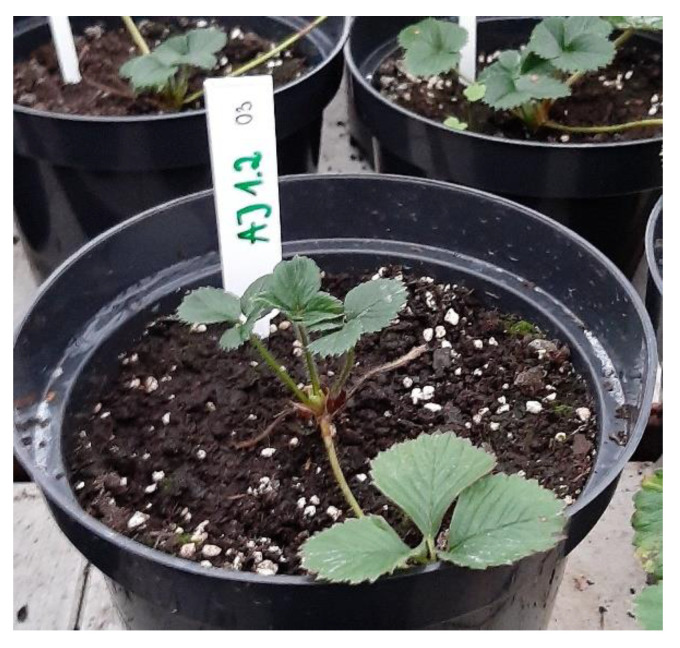
Strawberry cv. Polka at the time of inoculation.

A two-factor experiment was performed in a complete randomization design with four replications, each represented by a single plant. The first experimental factor was the inoculation of plant roots with rhizospheric bacteria. The inoculum was applied to the growth substrate near the root system, in the amount of 40 cm^3^/plant, with a minimum bacterial density of 10^7^ CFU/g, within 7 weeks from planting the plants (BBCH16, Figure 12). The following variants of the first factor were applied:

− K0—plants not inoculated with rhizospheric bacteria,
− KMg—application of MgSO_4_ nutrient solution without bacteria to the growth substrate in the amount of 40 cm^3^/plant, +
− DLGB 2—inoculation with *Bacillus* sp. strain DLGB 2,
− DLGB 3—inoculation with *Bacillus* sp. strain DLGB 3,
− DKB 26—inoculation with *Bacillus* sp. strain DKB 26,
− DKB 58—inoculation with *Bacillus* sp. strain DKB 58,
− DKB 84—inoculation with *Bacillus* sp. strain DKB 84,
− DKB 63—inoculation with *Pantoea* sp. strain DKB 63,
− DKB 64—inoculation with *Pantoea* sp. strain DKB 64,
− DKB 65—inoculation with *Pantoea* sp. strain DKB 65,
− DKB 68—inoculation with *Pantoea* sp. strain DKB 68,
− DKB 70—inoculation with *Pantoea* sp. strain DKB 70,
− AJ 1.1—inoculation with *Azotobacter* sp. strain AJ 1.1,
− PJ 1.2—inoculation with *Pseudomonas* sp. strain PJ 1.2.

The *Bacillus* sp. and *Pantoea* sp. inocula came from the Department of Microbiology and Rhizosphere, the National Institute of Horticultural Research in Skierniewice (Poland); the strains of *Azotobacter* sp. and *Pseudomonas* sp. came from the Institute of Marine and Environmental Sciences of the University of Szczecin (Poland).

The second experimental factor was the different moisture content of the growth substrate. The water potential was maintained at −10 to −15 kPa under control conditions (optimal soil moisture—variant OM), and at −40 to −45 kPa under conditions of water deficit in the substrate (variant WD). The substrate moisture levels were varied from 6 weeks after inoculation. The substrate moisture was determined using soil contact tensiometers.

### 4.3. Bioassays for Plant Growth Promoting Traits

The mobilization of P from insoluble phosphate was detected by the formation of a transparent halo zone surrounding bacterial colonies on the Pikovskaya medium containing tricalcium phosphate, after 5 days at 28 °C [84]. Siderophore production was detected by the production of an orange halo zone on a standard Chrome Azurol-S (CAS) agar plate after 5 days at 28 °C [85,86].

The quantification of indole-3-acetic acid production was performed with Salkowski’s reagent [87]. Bacterial cultures were grown on minimal DF solid medium [88] supplemented with tryptophan (500 µg·mL^−1^) as the precursor of IAA. The plates were covered with Whatman No. 1 filter paper saturated with Salkowski’s reagent for 30 min. at 28 °C. A pink zone appeared around the IAA-producing colonies.

ACC deaminase activity was determined by a modified method that measures the amount of α-ketobutyrate (α-KB) when the ACC deaminase enzyme cleaves ACC. The bacterial strains were propagated in a minimal DF medium with 5 mM ACC. The calibration curve was formed on α-ketobutyrate. The ACCD activity was expressed as nM of α-KB·mg protein^−1^·h^−1^ [89,90].

### 4.4. Bacterial Counts in Substrate

Two grams of each substrate sample was added to 18 mL of 0.9% (*w*/*v*) solution of sodium chloride. After homogenization for 1h, this solution was decimally diluted (10^−2^ to 10^−6^), and 100 µL aliquots of the resulting solutions were plated on Tryptone Soya Agar (TSA, Oxoid). After incubation at 28 °C for 3 days, the colony forming units (CFU) were counted. The bacterial counts were performed on substrate samples taken from each pot on 12 April 2021, 18 weeks after the inoculation of the plants.

### 4.5. Chlorophyll “a” Fluorescence

The measurements of direct chlorophyll fluorescence were recorded using a Handy PEA (Handy Plant Efficiency Analyzer) spectrofluorometer (Hansatech Instruments Ltd., King’s Lynn, Norfolk, UK), based on the standard apparatus procedure (3 × 650 nm LEDs, maximum actinic light intensity 3500 μmol·m^−2^·s^−1^, duration of the light pulse 1s). The leaves were shaded for 20 min. prior to the measurement with a leaf clip (4 mm in diameter). The following parameters of chlorophyll fluorescence induction were measured and calculated using the spectrofluorometer: the index of initial fluorescence excitation energy loss in power antennas (F_0_); maximum fluorescence after the reduction in acceptors in PSII and after dark adaptation (F_M_); variable fluorescence, determined after dark adaptation, a parameter dependent on the maximum quantum yield of PSII (F_V_ = F_M_ − F_0_); maximum potential photochemical reaction efficiency in PSII determined after dark adaptation and after the reduction in acceptors in PS II (F_V_/F_M_); the time of fluorescence increase to the value of F_M_ (T_FM_); PSII vitality index for the overall viability of this system (PI); the surface area above the chlorophyll fluorescence curve and between the F_0_ and F_M_ points proportional to the size of the reduced plastoquinone acceptors in PS II (Area) [91]. The measurements of chlorophyll fluorescence parameters were taken 18 weeks after the inoculation of the plants, on healthy, fully grown leaves of each plant.

### 4.6. Statistical Methods

The results of the tests were subjected to bivariate analysis of variance (ANOVA), and Tukey’s HDS post hoc test was performed. Additionally, in order to determine the occurrence of the variability of the determined traits depending on the experimental factors used, a cluster analysis was carried out using Ward’s method for linkage and the square of the Euclidean distance as a measure of distance [92]. The significance of the clusters was determined using the Sneath graded criterion [93]. The above statistical calculations were performed using Statistica 13.1PL (Cracow, Poland, StatSoft Poland). A Monte Carlo simulation with R Studio software (Boston, USA, RStudio PBA) was performed to confirm the obtained results [94].

## 5. Conclusions

The presented studies in this paper on strawberry plants showed that the greatest usefulness for characterizing water stress was demonstrated by F_M_, F_V_, F_V_/F_M_, PI, and Area. Based on the assessment of the condition of the photosynthetic apparatus and the analysis of chlorophyll “a” fluorescence indices, the most favorable effects on strawberry plants under water deficit had the *Bacillus* sp. strains, DLGB2 and DKB26 and the *Pantoea* sp. strains, DKB63, DKB70, DKB68, DKB64, and DKB65. In the case of inoculation with the *Pantoea* sp. strain, DKB65 and *Bacillus* sp. strain, DLGB 2, the tests demonstrated the lowest decrease, among those recorded under water deficit, in the numbers of bacteria in the soil. In contrast, the *Pantoea* sp. strain, DKB64, was one of those that produced, after inoculation under water deficit, the highest number of bacteria. The recently ongoing climatic changes force us to look for sustainable and effective solutions to alleviate the water deficit stress in cultivated plants. Therefore, it is justified and necessary to conduct further, much wider research on the most promising beneficial microorganisms that favorably affect plants under stress conditions and alleviate the negative effects of water stress.

## Figures and Tables

**Table 1:** Some of the key traits of plant growth-promoting bacteria strains.

Bacteria Strain	PGPR Traits			
	IAA Production	Siderophore Production	Phosphate Solubilization	ACCD Activity (nmol α- KB·mg Protein^−1^·h^−1^)
AJ 1.1	+	+	+	8306.25 ± 114.2
PJ 1.2	+	+	+	6287.5 ± 122.4
DLGB 2	-	+	-	nd
DLGB 3	-	+	-	nd
DKB 26	-	+	-	853.0 ± 25.7
DKB 58	-	+	-	2946.75 ± 108.6
DKB 84	-	+	-	2742.0 ± 46.0
DKB 64	+	-	+	3862.25 ± 34.5
DKB 65	+	-	+	4757.0 ± 75.7
DKB 70	+	-	+	62.5 ± 29.2
DKB 63	-	-	+	107.0 ± 17.3
DKB 68	-	-	+	122.75 ± 23.7

-, no activity; +, activity; nd, not detected.

**Table 2:** Descriptive statistics results.

Variant	Descriptive Statistics	T_FM_		Area		F_0_	
		OM	WD	OM	WD	OM	WD
K0	Mean ± SD	837.5 ± 16.06	858.33 ± 11.86	69,490.33 ± 28	61,215.92 ± 19.76	230.63 ± 10.61	232.38 ± 14.6
	Range	400–900	500–900	29,086–98,646	30,210–89,071	202–312	202–371
	CV	16%	12%	28%	20%	11%	15%
KMg	Mean ± SD	891.67 ± 4.58	879.17 ± 6.69	72,806.5 ± 19.45	66,549.96 ± 18.08	222.08 ± 5.84	224.33 ± 12.27
	Range	700–900	700–900	53,216–10,2457	29,279–89,552	202–255	202–343
	CV	5%	7%	19%	18%	6%	12%
AJ 1.1	Mean ± SD	866.67 ± 10.24	875 ± 5.17	70,891.58 ± 23.05	63,561 ± 21.7	234.08 ± 10.7	226.42 ± 6.3
	Range	600–900	800–900	32,248–86,547	41,521–81,726	216–308	205–253
	CV	10%	5%	23%	22%	11%	6%
PJ 1.2	Mean ± SD	833.33 ± 17.97	891.67 ± 3.24	64,828 ± 24.39	64,587.67 ± 19.24	230.5 ± 5.11	221.83 ± 7.01
	Range	400–900	800–900	42,926–98,793	38,747–90,909	213–254	209–266
	CV	18%	3%	24%	19%	5%	7%
DLGB 2	Mean ± SD	841.67 ± 16.38	875 ± 7.1	72,136.58 ± 25.08	68,535.83 ± 8.81	244.67 ± 22.69	216.17 ± 5.3
	Range	500–900	700–900	31,493–97,428	61,022–78,161	193–376	195–231
	CV	16%	7%	25%	9%	23%	5%
DLGB 3	Mean ± SD	841.67 ± 17.88	883.33 ± 4.41	72,116.33 ± 26.93	71,489.42 ± 9.75	227.67 ± 13.27	222.67 ± 7.86
	Range	400–900	800–900	27,371–97,196	60,365–83,037	200–283	205–269
	CV	18%	4%	27%	10%	13%	8%
DKB 26	Mean ± SD	883.33 ± 4.41	883.33 ± 4.41	71,094.58 ± 24.12	68,786.42 ± 15.62	230.83 ± 3.2	218.08 ± 5.2
	Range	800–900	800–900	35,714–100,350	49,304–82,578	219–245	197–234
	CV	4%	4%	24%	16%	3%	5%
DKB 58	Mean ± SD	900 ± 0	883.33 ± 4.41	77,358.58 ± 16.48	68,580.42 ± 12.6	234.08 ± 10.91	217.75 ± 3.36
	Range	900–900	800–900	60,224–96,866	49,448–79,824	207,308	201,229
	CV	0%	4%	16%	13%	11%	3%
DKB 84	Mean ± SD	883.33 ± 4.41	900 ± 0	72,272.75 ± 16.14	67,263 ± 17.59	234.08 ± 14.62	222.08 ± 5.58
	Range	800–900	900–900	57,317–91,807	49,923–89,070	205–339	202–241
	CV	4%	0%	16%	18%	15%	6%
DKB 64	Mean ± SD	858.33 ± 11.61	900 ± 0	68,943.67 ± 23.86	75,019.58 ± 8.28	233.25 ± 8.98	211.92 ± 4.06
	Range	600–900	900–900	31,718–94,651	60,181–82,864	197–271	194–223
	CV	12%	0%	24%	8%	9%	4%
DKB 65	Mean ± SD	900 ± 0	891.67 ± 3.24	72,762.5 ± 10.73	78,063.92 ± 20.74	215.08 ± 2.63	219.33 ± 3.92
	Range	900–900	800–900	61,066–84,065	55,787–10,7411	206–225	205–234
	CV	0%	3%	11%	21%	3%	4%
DKB 70	Mean ± SD	872.73 ± 5.35	890.91 ± 3.38	71,595.27 ± 21.35	73,844.55 ± 14.44	234.64 ± 9.12	213.45 ± 4.35
	Range	800–900	800–900	39,133–90,337	52,905–87,403	214–285	194–226
	CV	5%	3%	21%	14%	9%	4%
DKB 63	Mean ± SD	883.33 ± 4.41	866.67 ± 7.52	71,595.25 ± 17.83	63,443.08 ± 30.34	229.83 ± 14.49	225.67 ± 23.01
	Range	800–900	700–900	48,060–94,376	17,269–91,168	206–332	159–373
	CV	4%	8%	18%	30%	14%	23%
DKB 68	Mean ± SD	866.67 ± 7.52	900 ± 0	70,508.58 ± 18.64	69,153.42 ± 27.11	228.17 ± 4.79	224.17 ± 14.5
	Range	700–900	900–900	47,235–91,287	25,942–96,307	209–244	203–324
	CV	8%	0%	19%	27%	5%	15%

**Table 3:** Descriptive statistics results.

Variant	Descriptive Statistics	F_M_		Fv		Fv/F_M_		PI	
		OM	WD	OM	WD	OM	WD	OM	WD
K0	Mean ± SD	1258.46 ± 11.01	1253.88 ± 7.26	1027.83 ± 13.88	1021.5 ± 10.08	0.81 ± 3.79	0.81 ± 4.24	10.18 ± 40.21	9.51 ± 36.46
	Range	984–1446	1038–1371	736–1217	777–1138	0.75–0.85	0.68–0.85	1.24–16.51	0.64–16.31
	CV	11%	7%	14%	10%	4%	4%	40%	36%
KMg	Mean ± SD	1254.5 ± 10.27	1258.25 ± 9.32	1032.42 ± 12.57	1033.92 ± 11.33	0.82 ± 2.71	0.82 ± 3.23	11.05 ± 25.62	11.29 ± 36.03
	Range	1023–1433	959–1462	795–1198	750–1237	0.78–0.85	0.72–0.85	5.74–15.94	0.74–17.16
	CV	10%	9%	13%	11%	3%	3%	26%	36%
AJ 1.1	Mean ± SD	1272.75 ± 9.3	1231.92 ± 9.13	1038.67 ± 12.32	1005.5 ± 11.53	0.81 ± 3.95	0.81 ± 2.81	8.94 ± 35.12	10.17 ± 36.19
	Range	1100–1429	1035–1405	814–1191	801–1186	0.73–0.84	0.77–0.84	1.13–12.24	5.78–17.07
	CV	9%	9%	12%	12%	4%	3%	35%	36%
PJ 1.2	Mean ± SD	1266.25 ± 10.66	1214.33 ± 10.96	1035.75 ± 13.39	992.5 ± 12.77	0.82 ± 3.01	0.82 ± 2.36	8.9 ± 32.49	10.78 ± 29.07
	Range	1040–1487	967–1369	810–1259	755–1146	0.78–0.85	0.78–0.84	5.59–13.61	4.97–15.3
	CV	11%	11%	13%	13%	3%	2%	32%	29%
DLGB 2	Mean ± SD	1286.08 ± 7.85	1237.08 ± 7.68	1041.42 ± 11.52	1020.92 ± 8.96	0.81 ± 5.93	0.82 ± 1.67	9.08 ± 46.01	12.73 ± 24.51
	Range	1112–1437	1091–1374	851–1200	867–1143	0.69–0.84	0.8–0.84	0.52–14.27	8.02–17.39
	CV	8%	0%	12%	9%	6%	2%	46%	25%
DLGB 3	Mean ± SD	1253.42 ± 8.83	1242.83 ± 7.91	1025.75 ± 12.1	1020.17 ± 10.31	0.82 ± 4.33	0.82 ± 3.11	11.29 ± 41.57	11.51 ± 31.15
	Range	1088–1384	1065–1350	806–1165	796–1135	0.74–0.85	0.75–0.84	3.06–17.72	3.74–16.03
	CV	9%	8%	12%	10%	4%	3%	42%	31%
DKB 26	Mean ± SD	1327.08 ± 7.45	1284.42 ± 10.08	1096.25 ± 9.11	1066.33 ± 11.92	0.83 ± 1.93	0.83 ± 2.2	9.08 ± 22.59	12.63 ± 26.6
	Range	1091–1454	1054–1450	856–1224	851–1230	0.79–0.84	0.79–0.85	3.78–12.25	6.8–19.67
	CV	7%	10%	9%	12%	2%	2%	23%	27%
DKB 58	Mean ± SD	1317.5 ± 7.21	1263.33 ± 6.15	1083.42 ± 9.66	1045.58 ± 7.36	0.82 ± 3.22	0.83 ± 1.39	9.95 ± 34.12	11.81 ± 16.31
	Range	1182–1461	1122–1393	937–1227	903–1176	0.75–0.85	0.81–0.84	3.03–14.78	7.56–14.65
	CV	7%	6%	10%	7%	3%	1%	34%	16%
DKB 84	Mean ± SD	1321.5 ± 6.86	1164 ± 12.14	1087.42 ± 9.99	941.92 ± 15.53	0.82 ± 4.27	0.81 ± 3.85	10.06 ± 34.33	10.3 ± 39.23
	Range	1116–1443	912–1376	867–1212	674–1156	0.72–0.84	0.74–0.85	1.94–15.89	3.03–16.52
	CV	7%	12%	10%	16%	4%	4%	34%	39%
DKB 64	Mean ± SD	1276.42 ± 10.22	1293.67 ± 4.72	1043.17 ± 13.13	1081.75 ± 5.78	0.82 ± 3.53	0.84 ± 1.33	8.66 ± 37.17	15.01 ± 16.9
	Range	1038–1469	1177–1378	813–1238	961–1163	0.77–0.85	0.82–0.85	4.5–14.4	10.07–19.1
	CV	10%	5%	13%	6%	4%	1%	37%	17%
DKB 65	Mean ± SD	1271.58 ± 7.67	1272.17 ± 5.07	1056.5 ± 9.28	1052.83 ± 6.08	0.83 ± 1.72	0.83 ± 1.27	12.49 ± 18.47	11.49 ± 24
	Range	1122–1395	1163–1348	916–1182	942–1128	0.81–0.85	0.81–0.84	8.56–16.52	6.33–15
	CV	8%	5%	9%	6%	2%	1%	18%	24%
DKB 70	Mean ± SD	1271.82 ± 9.05	1219.91 ± 10.61	1037.18 ± 11.72	1006.45 ± 12.43	0.81 ± 3.24	0.82 ± 2.06	8.63 ± 28.29	13.16 ± 21.64
	Range	1058–1425	952–1398	837–1193	758–1176	0.77–0.84	0.8–0.85	5.4–12.84	8.13–18.44
	CV	9%	11%	12%	12%	3%	2%	28%	22%
DKB 63	Mean ± SD	1294.25 ± 9.57	1189.25 ± 16.63	1064.42 ± 12.34	963.58 ± 21.53	0.82 ± 4	0.8 ± 8.39	10.66 ± 36.75	11.94 ± 43.75
	Range	1070–1458	779–1426	844–1234	554–1198	0.74–0.85	0.6–0.84	3.23–15.69	0.27–18.92
	CV	10%	17%	12%	22%	4%	8%	37%	44%
DKB 68	Mean ± SD	1276.75 ± 10.35	1183 ± 10.26	1048.58 ± 12.96	958.83 ± 14.72	0.82 ± 3.03	0.81 ± 5.86	10.4 ± 29.29	11.56 ± 49.21
	Range	994–1449	970–1342	756–1221	646–1130	0.76–0.85	0.67–0.84	4.53–13.88	1.02–23.96
	CV	10%	10%	13%	15%	3%	6%	29%	49%

**Table 4:** ANOVA results.

Variable	Main Effect				
	Sum of Squares	df	Mean Square Error	F	*p*
T_FM_	18,984	29	5539	3.42725	0.064900
Area	958,826,171	29	200,351,660	4.78572	0.029302
F_0_	6257	29	600	10.43421	0.001344
F_M_	141,911	29	13,807	10.27838	0.001459
F_V_	88,573	29	14,966	5.91849	0.015441
F_V_/F_M_	0.00007	29	0.0009	0.08489	0.770939
PI	220	29	13	16.75891	0.000052

## Data Availability

Data stored on the PTBD BIODATA server and in the public repository: https://github.com/PTBDBIODATA/Databases/blob/main/Fluoro%20DB.csv.
